# Modulation of Photosystem II Function in Celery via Foliar-Applied Salicylic Acid during Gradual Water Deficit Stress

**DOI:** 10.3390/ijms25126721

**Published:** 2024-06-18

**Authors:** Michael Moustakas, Emmanuel Panteris, Julietta Moustaka, Tuğba Aydın, Gülriz Bayçu, Ilektra Sperdouli

**Affiliations:** 1 Department of Botany, School of Biology, Aristotle University of Thessaloniki, 54124 Thessaloniki, Greece; moustak@bio.auth.gr (M.M.); epanter@bio.auth.gr (E.P.); 2 Department of Food Science, Aarhus University, 8200 Aarhus, Denmark; julietta_moustaka@food.au.dk; 3 Department of Biology, Faculty of Science, Istanbul University, 34134 Istanbul, Turkey; taydin1@ogr.iu.edu.tr (T.A.); gulrizb@istanbul.edu.tr (G.B.); 4 Institute of Plant Breeding and Genetic Resources, Hellenic Agricultural Organisation–Demeter (ELGO-Dimitra), 57001 Thermi, Greece

**Keywords:** drought, chloroplast ultrastructure, photoprotective heat dissipation, chlorophyll fluorescence imaging, electron transport rate, photochemical quenching, singlet oxygen

## Abstract

Water deficit is the major stress factor magnified by climate change that causes the most reductions in plant productivity. Knowledge of photosystem II (PSII) response mechanisms underlying crop vulnerability to drought is critical to better understanding the consequences of climate change on crop plants. Salicylic acid (SA) application under drought stress may stimulate PSII function, although the exact mechanism remains essentially unclear. To reveal the PSII response mechanism of celery plants sprayed with water (WA) or SA, we employed chlorophyll fluorescence imaging analysis at 48 h, 96 h, and 192 h after watering. The results showed that up to 96 h after watering, the stroma lamellae of SA-sprayed leaves appeared dilated, and the efficiency of PSII declined, compared to WA-sprayed plants, which displayed a better PSII function. However, 192 h after watering, the stroma lamellae of SA-sprayed leaves was restored, while SA boosted chlorophyll synthesis, and by ameliorating the osmotic potential of celery plants, it resulted in higher relative leaf water content compared to WA-sprayed plants. SA, by acting as an antioxidant under drought stress, suppressed phototoxicity, thereby offering PSII photoprotection, together with enhanced effective quantum yield of PSII photochemistry (Φ*_PSII_*) and decreased quantity of singlet oxygen (^1^O_2_) generation compared to WA-sprayed plants. The PSII photoprotection mechanism induced by SA under drought stress was triggered by non-photochemical quenching (NPQ), which is a strategy to protect the chloroplast from photo-oxidative damage by dissipating the excess light energy as heat. This photoprotective mechanism, triggered by NPQ under drought stress, was adequate in keeping, especially in high-light conditions, an equal fraction of open PSII reaction centers (q*p*) as of non-stress conditions. Thus, under water deficit stress, SA activates a regulatory network of stress and light energy partitioning signaling that can mitigate, to an extent, the water deficit stress on PSII functioning.

## 1. Introduction

Anthropogenic activities intensify the negative consequences of climate change that compromise sufficient food production [1]. It is estimated that global food production may be reduced by 11–25% at the end of this century [2]. Water deficit is the major stress factor magnified by climate change that causes the most reductions in productivity when compared to other stress factors [3,4,5,6,7,8]. The increasing demand for sustainable agricultural solutions has brought biostimulants to the forefront as valuable tools for regenerative farming [9,10]. Biostimulants, with their varied biological activities, play a significant role in enhancing crop growth, improving nutrient efficiency, sustaining resilience to environmental stressors, and restoring soil health [10].

Salicylic acid (SA) is considered an important phytohormone, being essential for controlling a broad array of biochemical and physiological processes that are involved in many stages of growth and development, including seed germination, flowering, regulation of ripening, and plant defense responses to pathogens and abiotic stimuli [11,12,13,14,15,16,17,18]. It has been suggested to have evident potential for mitigating numerous abiotic stresses of major agricultural crops, for improving their stress resistance, acting as an antioxidant, osmo-regulator, and biostimulant [17,19,20,21].

SA biosynthesis in plants has been described to start (i) in the chloroplast, where chorismate is converted to isochorismate via the isochorismate pathway and then it is transported to the cytosol, or (ii) with the amino acid L-phenylalanine in the cytosol, via the phenylalanine ammonia-lyase pathway [22]. However, in Arabidopsis, it has been shown that SA biosynthesis is not from the phenylalanine ammonia-lyase pathway [23] but it remains unclear whether this pathway is involved in other plant species [20].

An increase in SA production and signaling during defense responses is associated with a simultaneous reduction in auxin biosynthesis and signaling, thereby coordinating defense and growth [20,24]. SA application can alleviate the negative effects of water deficit and salinity by improving osmotic potential, reducing membrane damage, regulating stomatal conductance and transpiration rate, restoring biochemical parameters and photosynthetic rates, and enhancing shoot and root biomass as well as nutrient uptake [25,26]. Nevertheless, the effects of exogenously applied SA on plant physiological processes under optimal growth conditions are contentious, with some studies reporting positive effects on plant growth while others highlight negative influences on various physiological processes [27]. For instance, spraying SA on corn and soybean increased photosynthetic rates, possibly due to enhanced enzyme activity related to CO_2_ uptake rather than increased stomatal opening [27,28]. Conversely, applying 0.5 mM of SA to maize plants decreased net photosynthesis and stomatal conductance under both low and high light conditions [29]. Yet, the same concentration provided protection against low-temperature damage [29] or mitigated the damaging effects of paraquat [30]. The exogenous application of SA mitigated the effects of mild water deficit in radish plants [31].

Water deficit stress results in stomatal closure, limiting CO_2_ assimilation and, thus, reducing carbon fixation, which results in excess light energy that cannot be used for photochemistry [6,32,33,34,35]. This excess light energy is prospective for injury if it is transferred to O_2_, producing photo-oxidative damage and reducing photosynthetic efficiency [36,37,38,39,40]. Thus, the efficient management of the excess light energy that cannot be assigned to photochemistry under water deficit stress is fundamental [35,36,37]. Plants can dissipate this excess excitation energy as heat, via the mechanism of non-photochemical quenching (NPQ), to protect the chloroplasts and especially photosystem II (PSII) from photo-oxidative damage [35,36,40]. Knowledge of the PSII response mechanisms underlying crop vulnerability to water deficit stress is critical to better understanding the consequences of climate change on crop plants [4,6,7,35]. Investigating PSII-adaptive strategies to water deficit should, thus, assist in fulfilling this knowledge gap. Photosynthetic light energy use efficiency is generally mentioned to govern crop yields [41,42,43,44]. One key approach to improve the light energy utilization efficiency is to boost the dissipation of excess excitation energy by the NPQ mechanism [45,46,47,48,49].

The exogenous application of SA can modulate the electron transport rate (ETR) in photosystem II (PSII), by the NPQ mechanism that dissipates the excess light energy as heat [27,50,51,52]. However, the mechanism of the SA-mediated response of PSII photochemistry under non-stress or drought stress conditions is diverse [51,52]. PSII photochemistry was reported to be enhanced by SA under non-stress conditions only under high light but under drought stress at both low light and high light conditions [52]. In addition, the SA mode of action has been described to differ between plant species [51,52,53]. These different effects of SA on distinct plant species can be attributed to variations in its biosynthesis and signaling pathways in different plants [22]. The plant species variety of the SA defense signaling is also apparent in SA accumulation, interplay with other hormones, and redox regulation [54].

Celery (*Apium graveolens* L.) is an important biennial vegetable crop, widely grown worldwide and dating back to the 7th century B.C. [55,56]. It originated in the Middle East and the Mediterranean area and now has a large geographical distribution and a long cultivation history [55,56]. It is used in medicine as an anti-inflammatory and antibacterial agent and in food and cosmetic industries [57,58]. Celery is considered a drought-tolerant species due to its antioxidant activities [56]. The exogenous application of proline has been shown to increase the salt tolerance of celery [59] while foliar fertilizers improve its yield and quality [60].

In our research work, we tested the hypothesis that the foliar application of SA would improve the light energy utilization efficiency in celery plants by boosting the dissipation of excess excitation energy via the NPQ mechanism, thus enhancing PSII function and improving drought tolerance. We hypothesize that SA-sprayed leaves under water deficit conditions would have an improved osmotic potential and would maintain a higher leaf water content that may enable an enhanced PSII function compared to WA-sprayed leaves with reduced leaf water content.

## 2. Results

### 2.1. Soil and Leaf Water Content under Treatments

Celery plants were watered, then foliar sprayed with water or SA once, while measurements were performed 48 h, 96 h, and 192 h after the spray. Forty-eight hours after watering, the soil water content of WA-sprayed plants decreased to 70 ± 3% of the full soil water capacity, while in SA-sprayed plants, the soil water content decreased to 82 ± 4% (Table 1). At the further elapsed time from watering, 96 h and 192 h, the soil water content of WA-sprayed plants decreased further to 27 ± 2% and 5 ± 1% of the full soil water capacity, respectively, while in the SA-sprayed plants, this decreased to 38 ± 3% and 9 ± 2%, respectively (Table 1).

The same trend was noticed in the leaf relative water content: at 48 h, 96 h, and 192 h after watering, in WA-sprayed plants, this content decreased to 81 ± 0.4%, 79 ± 0.2%, and 73 ± 0.1%, respectively; meanwhile, in the SA-sprayed plants, this decreased to 85 ± 0.3%, 83 ± 0.3%, and 80 ± 0.2%, respectively (Table 1).

### 2.2. Chlorophyll Content in Water-Sprayed and Salicylic Acid-Sprayed Plants

Chlorophyll content in WA-sprayed leaves 48 h after watering was higher compared to SA-sprayed leaves but at 96 h after watering, chlorophyll content was higher in SA-sprayed leaves (Table 1). At the water deficit conditions, 192 h after watering, an amplification of chlorophyll content was observed in SA-sprayed plants while in WA-sprayed plants, chlorophyll content decreased to the lowest level (Table 1).

### 2.3. Light Energy Partitioning in Water-Sprayed and Salicylic Acid-Sprayed Plants

**Figure 1:**
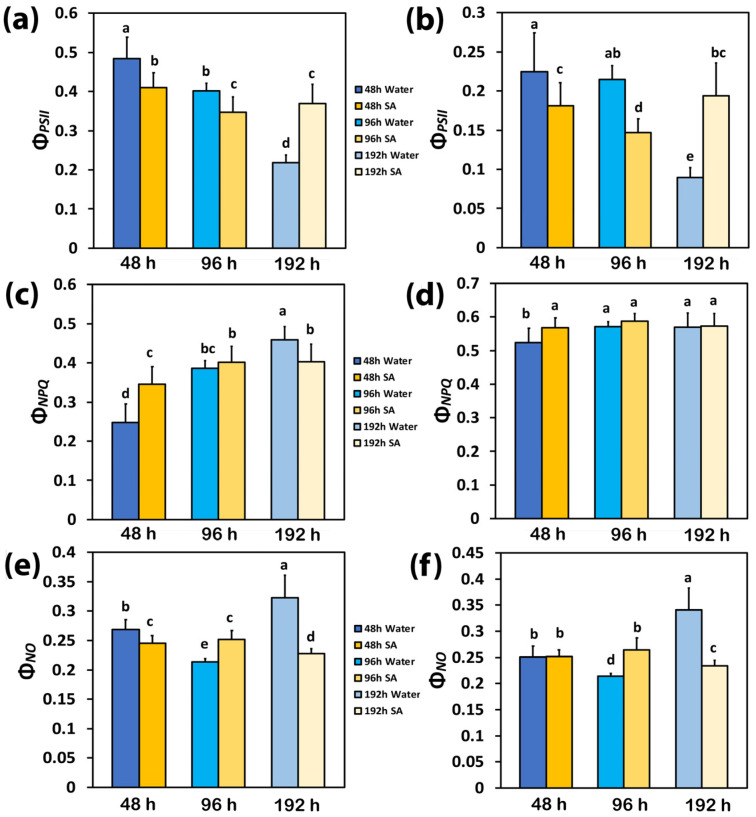
Light energy partitioning at PSII. The effective quantum yield of PSII photochemistry (Φ*_PSII_*) at low light (LL) (**a**) and at high light (HL) (**b**); the quantum yield of regulated non-photochemical energy loss in PSII (Φ*_NPQ_*) at LL (**c**) and at HL (**d**); and the quantum yield of non-regulated energy loss in PSII (Φ*_NO_*) at LL (**e**) and at HL (**f**) of water-sprayed or salicylic acid-sprayed (SA), celery plants at 48 h, 96 h, and 192 h after watering. Standard deviations (SD) are shown as error bars (n = 9, 3 independent experiments with 3 plants for each treatment and time point measurement). Significant differences are expressed by different alphabet letters.

The yield for photochemistry (Φ*_PSII_*) and the yield losses, regulated (Φ*_NPQ_*), and non-regulated (Φ*_NO_*), have a sum equal to 1 [61]. Forty-eight hours after watering, Φ*_PSII_* in WA-sprayed leaves was significantly higher than in SA-sprayed leaves at both low light (LL) (Figure 1a) and high light (HL) (Figure 1b). At 96 h after watering, Φ*_PSII_* decreased in SA-sprayed leaves under both LL (Figure 1a) and HL (Figure 1b) but in WA-sprayed leaves, Φ*_PSII_* decreased only under LL (Figure 1a). At the prolonged time from watering (192 h, water deficit conditions), Φ*_PSII_* in WA-sprayed leaves decreased to the lowest level under both LL (Figure 1a) and HL (Figure 1b); meanwhile, in SA-sprayed leaves at 192 h after watering, Φ*_PSII_* at LL remain the same to that of 96 h after watering (Figure 1a) and at HL, to that of 48 h after watering (Figure 1b).

At 48 h after watering, the decreased yield for photochemistry (Φ*_PSII_*) in SA-sprayed plants, compared to WA-sprayed ones (Figure 1a), was overcompensated at LL by the increased yield of regulated non-photochemical energy loss in PSII (Φ*_NPQ_*) (Figure 1c), which resulted in a lower yield of non-regulated energy loss in PSII (Φ*_NO_*) (Figure 1e). At 48 h after watering at HL, the increased Φ*_NPQ_* in SA-sprayed leaves, compared to WA-sprayed ones (Figure 1d), resulted in the same level of Φ*_NO_* with the WA-sprayed ones (Figure 1f) despite its lower Φ*_PSII_* (Figure 1b).

At 96 h after watering, the decreased Φ*_PSII_* in WA-sprayed plants (Figure 1a), compared to WA-sprayed plants at 48 h after watering, was overcompensated at LL by the increased yield of Φ*_NPQ_* (Figure 1c), which resulted in reduced Φ*_NO_* compared to WA-sprayed plants at 48 h after watering (Figure 1e). At 96 h after watering at HL, the increased Φ*_NPQ_* in WA-sprayed leaves (Figure 1d) developed reduced Φ*_NO_* compared to WA-sprayed plants at 48 h after watering (Figure 1f).

At 192 h after watering, the lowest level of Φ*_PSII_* in WA-sprayed leaves under both LL (Figure 1a) and HL (Figure 1b) could not be compensated by the increased Φ*_NPQ_* (Figure 1c,d); thus, the highest levels of Φ*_NO_* were developed (Figure 1e,f). In contrast, in SA-sprayed leaves, the increased Φ*_NPQ_* at both LL and HL (Figure 1c,d) could decrease Φ*_NO_* to lower levels than those observed in SA-sprayed leaves at 48 h after watering (Figure 1e,f).

### 2.4. The Photoprotective Heat Dissipation and the Electron Transport Rate in Water-Sprayed and Salicylic Acid-Sprayed Plants

**Figure 2:**
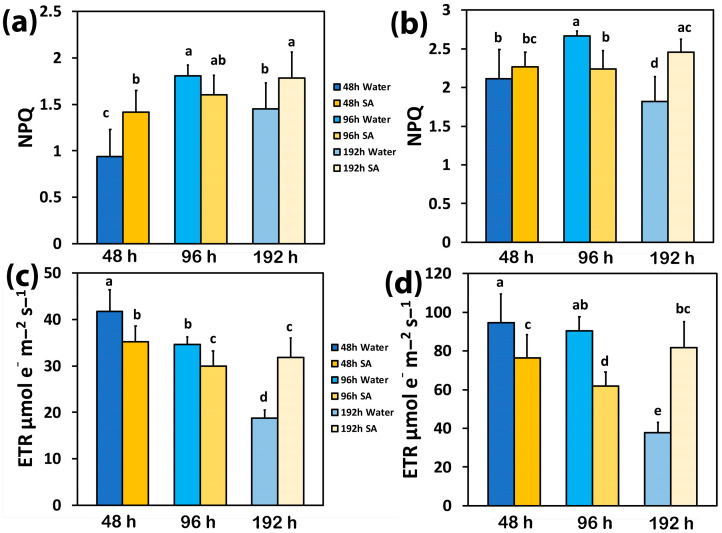
The non-photochemical quenching (NPQ) at low light (LL) (**a**) and at high light (HL) (**b**) and the electron transport rate (ETR) at LL (**c**) and at HL (**d**) of water-sprayed or salicylic acid-sprayed (SA) celery plants at 48 h, 96 h, and 192 h after watering. Standard deviations (SD) are shown as error bars (n = 9, 3 independent experiments with 3 plants for each treatment and time point measurement). Significant differences are expressed by different alphabet letters.

The photoprotective heat dissipation, via non-photochemical quenching (NPQ), increased with elapsed time from watering in both WA-sprayed and SA-sprayed leaves at both LL and HL (Figure 2a,b). The only exceptions were the WA-sprayed leaves at 192 h under both LL and HL, at which NPQ decreased, and the SA-sprayed leaves at 96 h under both LL and HL, at which NPQ remained at the level of 48 h after watering (Figure 2a,b).

Forty-eight hours after watering, the electron transport rate (ETR) in WA-sprayed leaves was significantly higher than in SA-sprayed leaves at both LL (Figure 2c) and HL (Figure 2d). At 96 h after watering, ETR decreased in SA-sprayed leaves under both LL and HL (Figure 2c,d) but in WA-sprayed leaves, ETR decreased only under LL (Figure 2c). Under water deficit conditions (192 h after watering), ETR decreased in WA-sprayed leaves to the lowest level under both LL (Figure 2c) and HL (Figure 2d); meanwhile, in SA-sprayed leaves at 192 h after watering, ETR remained at the same level as at 96 h after watering at LL (Figure 2c) and at that of 48 h after watering at HL (Figure 2d).

### 2.5. The Fraction of Open PSII Reaction Centers and Their Efficiency in Water-Sprayed and Salicylic Acid-Sprayed Plants

**Figure 3:**
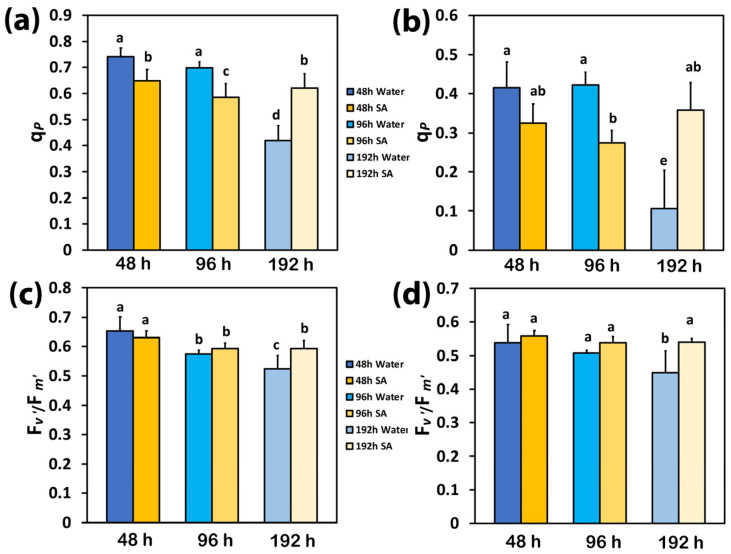
The photochemical quenching (q*p*), representing the fraction of open PSII reaction centers (RCs) at low light (LL) (**a**) and at high light (HL) (**b**), and the efficiency of the open PSII RCs (F*v*′/F*m*′) at LL (**c**) and at HL (**d**) of water-sprayed or salicylic acid-sprayed (SA) celery plants at 48 h, 96 h, and 192 h after watering. Standard deviations (SD) are shown as error bars (n = 9, 3 independent experiments with 3 plants for each treatment and time point measurement). Significant differences are expressed by different alphabet letters.

The redox state of quinone A (Q_A_), or the photochemical quenching (q*p*), also representing the fraction of open PSII reaction centers (RCs), was significantly higher in WA-sprayed leaves compared to SA-sprayed leaves at 48 h and 96 h after watering at both LL (Figure 3a) and HL (Figure 3b). However, at 192 h after watering, a higher portion of open PSII RCs was observed in SA-sprayed leaves compared to WA-sprayed ones at both LL (Figure 3a) and HL (Figure 3b). Under both LL and HL, WA-sprayed leaves possessed the lowest fraction of open PSII RCs while SA-sprayed leaves could retain the same fraction of open PSII RCs to that observed at 48 h after watering (Figure 3a,b).

The efficiency of the open PSII RCs (F*v*′/F*m*′) at both LL and HL did not differ between WA-sprayed and SA-sprayed leaves at 48 h and 96 h after watering (Figure 3c,d). However, at 192 h after watering, a higher efficiency of the open PSII RCs was observed in SA-sprayed leaves at both LL (Figure 3c) and HL (Figure 3d). At 192 h after watering at HL, the efficiency of the open PSII RCs in SA-sprayed leaves was similar to that at 48 h after watering (Figure 3d) while at LL, it was similar to that at 96 h after watering (Figure 3c).

### 2.6. Excess Excitation Energy and PSII Excitation Pressure in Water-Sprayed and Salicylic Acid-Sprayed Plants

**Figure 4:**
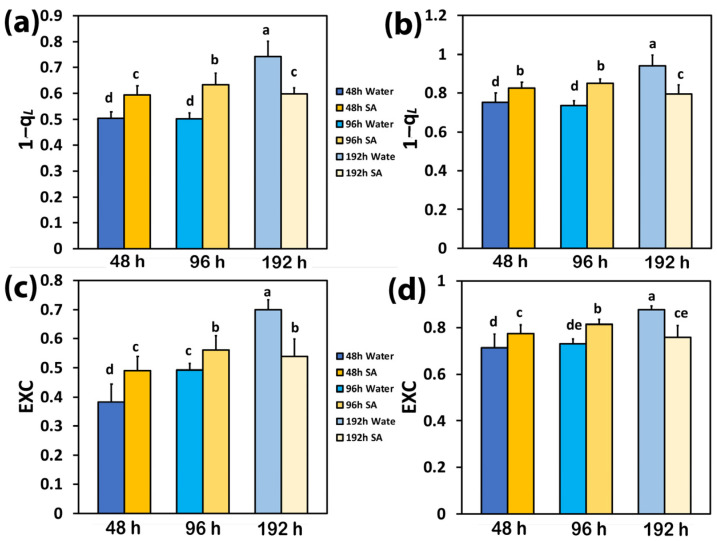
The excitation pressure at PSII (1−*qL*) at low light (LL) (**a**) and at high light (HL) (**b**) and the excess excitation energy at PSII (EXC) at LL (**c**) and at HL (**d**) of water-sprayed or salicylic acid-sprayed (SA) celery plants at 48 h, 96 h, and 192 h after watering. Standard deviations (SD) are shown as error bars (n = 9, 3 independent experiments with 3 plants for each treatment and time point measurement). Significant differences are expressed by different alphabet letters.

At 48 h and 96 h after watering, the excitation pressure at PSII (1−*qL*) in SA-sprayed leaves was higher at both LL (Figure 4a) and HL (Figure 4b) compared to WA-sprayed leaves; but at 192 h after watering, the excitation pressure (1−*qL*) at both LL (Figure 4a) and HL (Figure 4b) was higher in WA-sprayed leaves.

The same trend with excitation pressure (1−*qL*) was observed in the excess excitation energy at PSII (EXC), which at both LL (Figure 4c) and HL (Figure 4d) was higher in SA-sprayed leaves, at both 48 h and 96 h after watering, compared to WA-sprayed leaves; but at 192 h after watering, the excess excitation energy (EXC) at both LL (Figure 4c) and HL (Figure 4d) was higher in WA-sprayed leaves.

### 2.7. The Spatiotemporal Heterogeneity of PSII Function in Water-Sprayed and Salicylic Acid-Sprayed Plants

**Figure 5:**
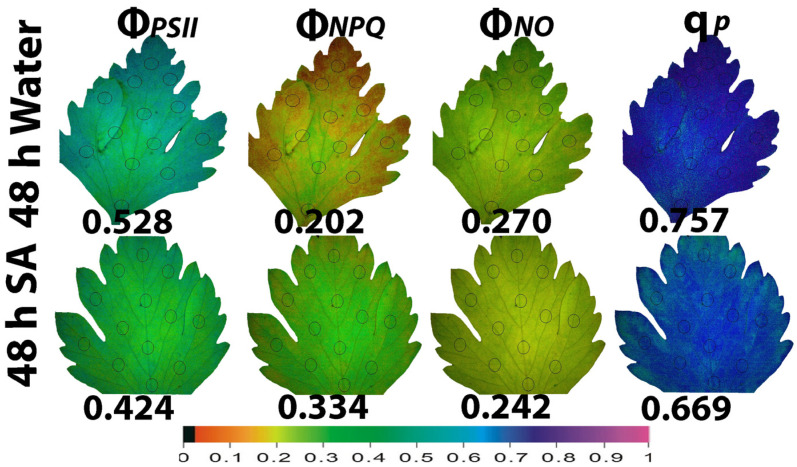
Whole leaf area color-coded pictures of Φ*_PSII_*, Φ*_NPQ_*, Φ*_NO_*, and q*p*, obtained at 200 μmol photons m^−2^ s^−1^, in water-sprayed and salicylic acid-sprayed (SA) celery plants at 48 h after watering. The average whole leaf value of each chlorophyll parameter is provided. At the bottom, the color code indicates the corresponding parameter value as color with a scale from 0 to 1.

**Figure 6:**
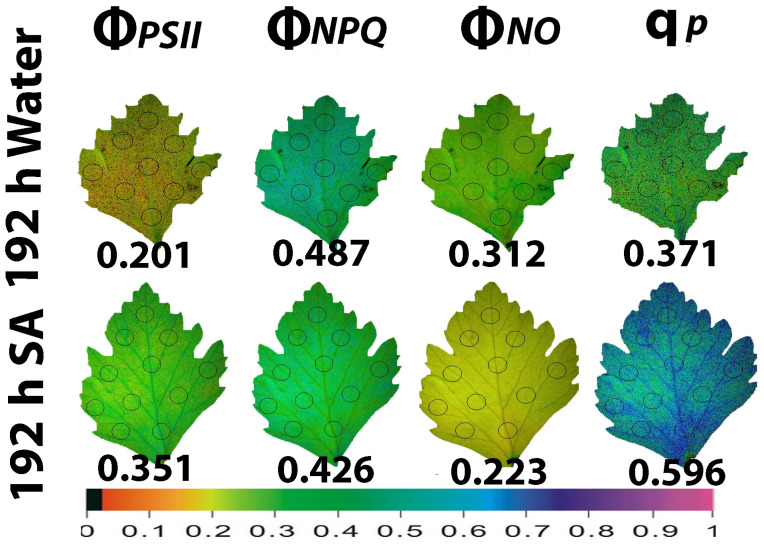
Whole leaf area color-coded pictures of Φ*_PSII_*, Φ*_NPQ_*, Φ*_NO_*, and q*p*, obtained at 200 μmol photons m^−2^ s^−1^, in water-sprayed and salicylic acid-sprayed (SA) celery plants at 192 h after watering. The average whole leaf value of each chlorophyll parameter is provided. At the bottom, the color code indicates the corresponding parameter value as color with a scale from 0 to 1.

The whole leaf area color-coded pictures of Φ*_PSII_*, Φ*_NPQ_*, Φ*_NO_*, and q*p* that were obtained with chlorophyll fluorescence imaging, at 48 h after watering (Figure 5) and at 192 h after watering (Figure 6), are presented. Forty-eight hours after watering, the yield for photochemistry (Φ*_PSII_*) decreased more in SA-sprayed leaves compared to WA-sprayed ones (Figure 5). However, a higher leaf heterogeneity of the parameter Φ*_PSII_* was observed in WA-sprayed leaves compared to SA-sprayed leaves (Figure 5). This higher leaf heterogeneity of the parameter Φ*_PSII_* in WA-sprayed leaves, compared to SA-sprayed ones, was noticed also for the parameter Φ*_NPQ_* (Figure 5). The higher increase in Φ*_NPQ_* in SA-sprayed leaves could overcompensate for the lower Φ*_PSII_* values compared to WA-sprayed leaves, resulting in decreased Φ*_NO_* values in SA-sprayed leaves (Figure 5). The higher Φ*_PSII_* values in WA-sprayed leaves also implied a higher fraction of open PSII RCs compared to SA-sprayed leaves (Figure 5). Thus, at 48 h after watering, the WA-sprayed leaves presented a better PSII functionality than SA-sprayed leaves.

At the prolonged time from watering (192 h, water deficit conditions), Φ*_PSII_* in WA-sprayed leaves was significantly lower than in SA-sprayed leaves (Figure 6). This decreased Φ*_PSII_* in WA-sprayed leaves could not be balanced by the increased Φ*_NPQ_* having as a result an increased Φ*_NO_* in WA-sprayed leaves, compared to SA-sprayed leaves (Figure 6). At 192 h after watering, SA-sprayed leaves also possessed a higher fraction of open PSII RCs compared to WA-sprayed leaves (Figure 6). Thus, at 192 h after watering, a better PSII functionality was observed in SA-sprayed leaves compared to WA-sprayed leaves (Figure 6). SA-sprayed leaves could maintain at 48 h (Figure 5) and at 192 h (Figure 6) after watering, lower Φ*_NO_* values compared to WA-sprayed leaves.

### 2.8. Chloroplast Ultrastructure in Water-Sprayed and Salicylic Acid-Sprayed Plants

**Figure 7:**
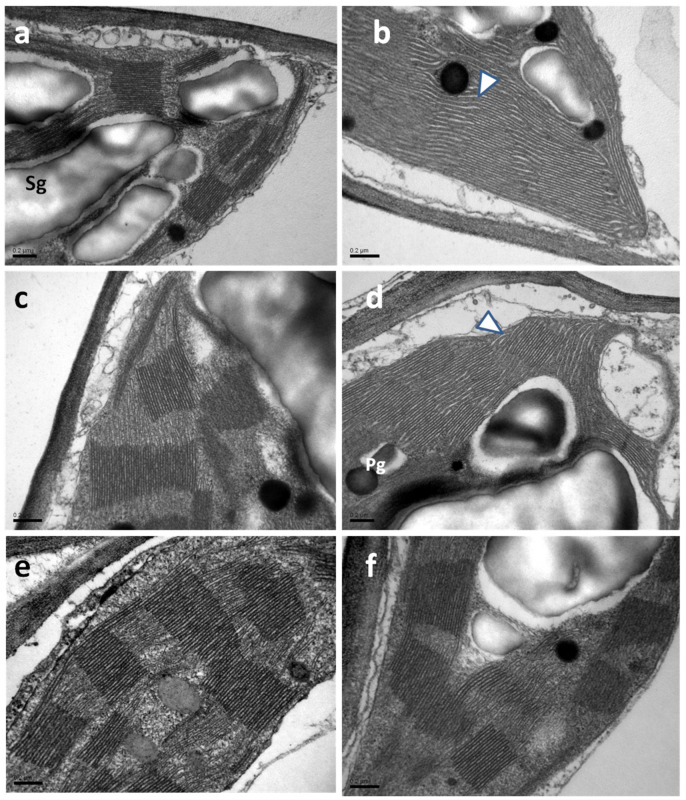
TEM micrographs depicting chloroplast ultrastructure in leaves of WA-sprayed (**a**,**c**,**e**) and SA-sprayed (**b**,**d**,**f**) plants at 48 h (**a**,**b**), 96 h (**c**,**d**), and 192 h (**e**,**f**) after watering. Note the dilated appearance of stroma lamellae in chloroplasts of SA-sprayed plants 48 h and 96 h after watering [arrowheads in (**b**,**d**)]. Pg: plastoglobuli, Sg: starch grain, scale bars: 0.2 μm.

Most of the typical ultrastructural features of chloroplasts, such as plastid envelope integrity, abundance of grana, starch grains, and plastoglobuli, were noticed in celery leaves, resembling those that are constantly observed by transmission electron microscopy (TEM) in leaf cells (Figure 7). However, in the chloroplasts of SA-sprayed celery leaves, at 48 h and 96 h after watering, the stroma lamellae appeared dilated (Figure 7b,d) in comparison with those observed in WA-sprayed leaves (Figure 7a,c). Interestingly, the above dilation was not observed 192 h after watering (water deficit conditions) in chloroplasts of SA-sprayed leaves, the stroma lamellae of which (Figure 7f) appeared normal, like those of WA-sprayed leaves at 48 h and 96 h after watering.

## 3. Discussion

Molecular mechanisms induced by biostimulants that can enhance osmolyte accumulation, e.g., proline, can counterbalance water loss and are efficient at maintaining high leaf water potential while soil water content is low [62,63,64]. Biostimulant-driven solutions can enhance crop drought tolerance and, thus, resolve a threatening agricultural challenge [64]. Many studies have demonstrated that the exogenous application of SA increases plant tolerance to drought stress by acting as an osmo-regulator [52,65]. This phenomenon is commonly referred to as osmotic adjustment, an essential physiological mechanism for drought tolerance [62,65]. In our experiment, at an increased time from watering, the soil water content in both WA-sprayed and SA-sprayed plants decreased, but SA-sprayed plants retained a higher percentage of soil water content under all time point measurements (Table 1). This was possible due to the reduced transpiration of SA-sprayed plants. In addition, SA-sprayed plants retaining a higher percentage of soil water content, they also maintained a higher leaf water content (Table 1).

At 48 h after watering, the chlorophyll content was higher in WA-sprayed leaves compared to SA-sprayed (Table 1). However, at 96 h and 192 h after watering, chlorophyll content enhanced in SA-sprayed leaves in a logarithmic-dependent manner to the leaf water loss (Table 1). A decrease in chlorophyll content under non-stress conditions and an increase under water deficit stress via the application of SA have been mentioned before [51,52]. Under deficit stress conditions (192 h after watering), chlorophyll content in WA-sprayed leaves diminished (Table 1). This may be ascribed to the oxidation of chlorophyll molecules in WA-sprayed leaves [66,67], which was reversed via the application of SA, which acted as an antioxidant in SA-sprayed leaves [52,68]. SA has been reported to act as a signal molecule activating chlorophyll catabolic genes [69]. An increasing or decreasing chlorophyll content through the application of SA linked to the plant species or even to the genotype [51,52,70,71,72] and/or to the concentration of SA used has been stated [73,74]. It has been frequently concluded that the mode of SA action varies considerably depending on the plant species, the genotype, the concentration used, the environmental conditions, and the duration of exposure [27,51,52,75,76].

Up to 96 h after watering, WA-sprayed celery leaves possessed a higher quantum yield of PSII photochemistry (Φ*_PSII_*) under both LL and HL compared to SA-sprayed leaves (Figure 1a,b). The dilation of stroma lamellae in the chloroplasts of SA-sprayed leaves (Figure 7b,d), but not in those of water-sprayed leaves (Figure 7a,c), could be an observable manifestation of the above difference. Thus, under non-stress conditions up to 96 h after watering, WA-sprayed celery leaves possessed higher Φ*_PSII_* values (Figure 1a,b) and ETR (Figure 2c,d) at both LL and HL compared to SA-sprayed celery leaves. These higher quantum yields of PSII photochemistry (Φ*_PSII_*) and electron transport rates (ETR) of WA-sprayed celery leaves were due to the higher fraction of open PSII RCs (Figure 3a,b) since the efficiency of the open PSII RCs (Figure 3c,d) did not differ between WA-sprayed and SA-sprayed leaves. In addition, WA-sprayed celery leaves, up to 96 h after watering, retained lower excess excitation energy at PSII (EXC) (Figure 4c,d) and lower excitation pressure (1−*qL*) (Figure 4a,b) compared to SA-sprayed leaves at both LL and HL. It seems that the delay in the enhancing response of PSII photochemistry to the SA spray was longer than 96 h.

WA-sprayed celery leaves 192 h after watering displayed the lowest quantum yield of PSII photochemistry (Φ*_PSII_*) under both LL and HL (Figure 1a,b) and the highest level of quantum yield of non-regulated energy loss in PSII (Φ*_NO_*), also under both LL and HL (Figure 1c,d). An increased Φ*_NO_* denotes an increased triplet chlorophyll state (^3^Chl*) population that results in the creation of singlet oxygen (^1^O_2_) [77,78,79]. Therefore, the possibility of ^1^O_2_ development can be calculated by Φ*_NO_* [79,80,81]. A decreased Φ*_NO_*, 192 h after watering in SA-sprayed celery leaves, compared to WA-sprayed ones (Figure 1c,d), reveals a lower ^1^O_2_ production [35,80,81,82] and suggests that SA acting as an antioxidant offers a better photoprotection. The lower fraction of open PSII reaction centers (q*p*) in WA-sprayed celery leaves 192 h after watering (Figure 3a,b), corresponding to a more reduced state of the plastoquinone (PQ) pool, was connected with a significant increase in ^1^O_2_ production (Figure 1c,d).

It is feasible that the increased generation of ^1^O_2_ production in WA-sprayed celery leaves 192 h after watering contributed to the increased photoinhibition of PSII [83]. Photoinhibition is described as an imbalance between PSII photodamage and PSII repair [84,85]. The photoprotective mechanism to prevent the photo-oxidative damage of excess excitation energy is the non-photochemical quenching (NPQ) [36,37,38,40,86]. The absorbed light energy that is not used for photochemistry, nor dissipated as heat for photoprotection via the NPQ mechanism, is the cause of PSII damage [87,88,89]. In WA-sprayed celery leaves 192 h after watering, the energy dissipation via NPQ (Figure 2a,b) was insufficient to prevent the over-excitation of the photosynthetic apparatus, resulting in increased ^1^O_2_ production (Figure 1c,d), which was supplemented by excess excitation energy at PSII (EXC) (Figure 4c,d) and increased excitation pressure (1−*qL*) (Figure 4a,b).

In contrast to WA-sprayed celery leaves—in which 192 h after watering, the ^1^O_2_ production increased—in SA-sprayed leaves, SA, acting as an antioxidant, decreased ^1^O_2_ production and enhanced the effective quantum yield of PSII (Φ*_PSII_*). The constructive role of SA under several environmental stress conditions is related to its capacity to reduce oxidative damage [68,90,91], serve as an antioxidant [51,52,68], and perform a controlling role in photosynthetic light reactions [92]. It is concluded that under deficit stress conditions, the high increase in NPQ in SA-sprayed leaves decreased ^1^O_2_ production, thereby inducing acclimation responses to water deficit stress. Singlet oxygen (^1^O_2_) is a reactive oxygen species (ROS) that can be developed in plants as a byproduct of photosynthesis [36,38,40,93]. ROS, such as superoxide anion radical (O_2_^•−^), hydrogen peroxide (H_2_O_2_), and singlet-excited oxygen (^1^O_2_), are constantly produced but are scavenged by the antioxidant enzymatic and non-enzymatic cellular mechanisms [40,94,95,96,97,98,99]. The function of chloroplast antioxidants is not to entirely remove ROS, but to accomplish a proper equilibrium between creation and removal so as to counterbalance the process of photosynthesis and document an efficient spread of signal wave [99,100,101,102]. ROS are fundamental signaling molecules that allow cells to respond speedily to diverse kinds of alterations to their homeostasis, contributing to the creation of defense mechanisms and plant resilience [103,104].

The photoprotective mechanism of NPQ is considered to be adequate, under stressful conditions, if it can retain an equal fraction of open PSII reaction centers (q*p*) as in non-stress conditions [4,99,105,106]. If not, an inconsistency between the absorbed light energy and the requirement occurs, indicating excess excitation energy [36,38,105]. The redox state of the PQ pool is recognized to be essential for retrograde signaling [107,108,109]. The fraction of open PSII reaction centers, or the redox state of the plastoquinone pool (q*p*), also involves a mechanism of plant acclimation to abiotic stresses by controlling the photosynthetic gene expression [98,110,111,112] and is of remarkable importance for antioxidant defense and signaling [113].

The induction of NPQ for preventing harmful ROS production, under environmentally stressful conditions [19,38,40,114], is connected with dynamic changes in protein–membrane association for regulating photosynthetic ETR [115]. It can be postulated that under water deficit conditions (192 h after watering), the high increase in NPQ in SA-sprayed leaves (Figure 2a,b), contributed to the restoration of the protein–membrane changes in the thylakoid structure (Figure 7f), thereby regulating the photosynthetic ETR of SA-sprayed leaves. SA protected PSII in *Arabidopsis thaliana* under HL by dissipating excess excitation energy and alleviating photoinhibition through the enhanced repair of the D1 protein [116]. Also, SA enhanced PSII efficiency in basil plants under non-stress conditions and improved photoprotection under mild drought stress by dissipating excess excitation energy [117].

To achieve a deeper understanding of SA-mediated defense networks and plant tolerance to environmental stresses, it is crucial to utilize a combination of plant physiology, molecular biology, computational biology, genomic, biochemistry, and bioinformatic approaches [118]. Future research based on these approaches can uncover the complex mechanisms behind SA-mediated defense pathways and their interactions with other signaling molecules for boosting plant stress resistance [118].

## 4. Materials and Methods

### 4.1. Plant Material and Growth Conditions

Celery (*Apium graveolens* L.) plants grown in standard potting soil medium were purchased from the “Garden Center Vaseiliadis” and transferred to a growth room with a light intensity of 200 ± 10 µmol photons m^−2^ s^−1^ and a 14 h photoperiod with a 21 ± 1/19 ± 1 °C day/night temperature and a relative humidity of 55 ± 5/65 ± 5% day/night.

### 4.2. Salicylic Acid Treatments

All celery plants were irrigated at full soil water capacity and then sprayed with 15 mL of either distilled water (WA, control) or with 1 mM of salicylic acid (SA) [51] one time. Measurements were performed 48 h, 96 h, and 192 h after the foliar spray of the well-watered plants. The experiment was executed with three independent repetitions, each with three plants per treatment and time point measurement.

### 4.3. Soil Volumetric Water Content

Soil volumetric water content was assessed using the soil moisture sensor (5TE; Decagon Devices, Pullman, WA, USA) combined with the readout device (ProCheck; Decagon Devices). The results of the volumetric soil water content measured in m^3^ m^−3^ were expressed as the percentage of the well-watered celery plants.

### 4.4. Leaf Water Content

The leaf water content of celery plants was evaluated using the electronic moisture balance (MOC120H, Shimadzu, Tokyo, Japan) as described previously [119].

### 4.5. Chlorophyll Content

The chlorophyll content of celery plants was expressed in relative units after being measured photometrically with a portable chlorophyll content meter (Model Cl-01, Hansatech Instruments Ltd., Norfolk, UK) [120].

### 4.6. Chlorophyll Fluorescence Imaging Analysis

Chlorophyll fluorescence imaging analysis was performed using the modulated Imaging-PAM Fluorometer M-Series (Heinz Walz GmbH, Effeltrich, Germany) as described in detail previously [121]. Celery plants were dark-adapted for 30 min before measurements that were conducted 48 h, 96 h, and 192 h after watering using the actinic light (AL) of 200 μmol photons m^−2^ s^−1^ (low light, LL) or 900 μmol photons m^−2^ s^−1^ (high light, HL). The chlorophyll fluorescence parameters that were assessed, using the Win software (Heinz Walz GmbH, Effeltrich, Germany), are described in Supplementary Table S1. Color-coded images of WA-sprayed and SA-sprayed celery plants that were recorded 48 h and 192 h after watering are presented.

### 4.7. Transmission Electron Microscopy

Leaves of plants treated as described in Section 4.2 were free-hand-cut in pieces measuring ~2 × 2 mm^2^, which underwent immediate fixation in 3% glutarhaldehyde in 50 mM sodium cacodylate buffer (pH 7) for 4 h at room temperature. After 3 rinses, 15 min each, in the same buffer, the samples were post-fixed overnight in 1% osmium tetroxide at 4 °C. After rinsing as above, the samples were gradually dehydrated in an acetone series, treated 2 × 20 min with propylenoxide at 4 °C, infiltrated, and finally embedded in Spurr’s resin. Ultrathin sections (~70 nm) were cut with a diamond knife and collected on copper grids. Four grids per treatment were examined. After double staining with uranyl acetate and lead citrate, the sections were examined at 80 kV with a JEOL JEM 1011 (JEOL, Tokyo, Japan) transmission electron microscope. Electron micrographs were acquired with a GATAN 500 digital camera (Gatan, Pleasanton, CA, USA).

### 4.8. Statistical Analysis

All statistical analyses were performed in R software, version 4.3.1 (R Core Team, 2023). The data were tested for normality and homogeneity of variance with the Shapiro–Wilk test and Levene’s test. When the assumptions were not met, a log transformation was used. Consequently, a two-way ANOVA was performed for each photosynthetic parameter with treatment (SA or WA) and time (48 h, 96 h, and 192 h) as factors, followed by a post hoc analysis with Tukey’s honest significant difference method with the R package ‘multcomp’. Values were considered significantly different at *p* < 0.05.

## 5. Conclusions

Our data show that SA-sprayed leaves under water deficit conditions (192 h after watering) had an improved osmotic potential and maintained a higher leaf water content that enabled an enhanced PSII function, compared to WA-sprayed leaves that retained lower leaf water content. Our hypothesis that SA would improve the light energy utilization efficiency in celery plants by boosting the dissipation of excess excitation energy via the NPQ mechanism, thus enhancing PSII function and improving drought stress tolerance, was confirmed. It seems that under water deficit stress, SA activates a regulatory network of stress and light energy partition signaling that can mitigate water deficit stress on the PSII function; but under non-stress conditions, at least in celery plants, it reduces PSII efficiency. SA-sprayed celery leaves were less efficient in utilizing light energy up to 96 h after watering compared to WA-sprayed leaves. This trend was reversed 192 h after watering (water deficit conditions) when SA induced the NPQ mechanism to protect the chloroplast from photo-oxidative damage. This was accomplished by dissipating the excess light energy as heat and, thus, restoring the drought stress-induced decrease in PSII efficiency. The drought resilience of PSII in SA-sprayed leaves was achieved, especially at HL conditions, through the ability of the SA-sprayed leaves to retain an equal fraction of open PSII RCs (q*p*) as in non-stress conditions.

## Figures and Tables

**Table 1:** Soil and leaf water content and chlorophyll content of celery plants under treatments.

Treatments	Soil Water Content (%) *	Relative Leaf Water Content (%)	Chlorophyll Content **
Water-sprayed plants after 48 h	70 ± 3%	81 ± 0.4%	6.14 ± 0.52
Water-sprayed plants after 96 h	27 ± 2%	79 ± 0.2%	7.11 ± 2.33
Water-sprayed plants after 192 h	5 ± 1%	73 ± 0.1%	2.82 ± 0.97
Salicylic acid-sprayed plants after 48 h	82 ± 4%	85 ± 0.3%	4.04 ± 1.12
Salicylic acid-sprayed plants after 96 h	38 ± 3%	83 ± 0.3%	12.80 ± 1.99
Salicylic acid-sprayed plants after 192 h	9 ± 2%	80 ± 0.2%	20.23 ± 4.99

* As a percentage of full soil water capacity; ** expressed in relative units; standard deviations are shown.

## Data Availability

The data presented in this study are available in this article.

## Supplementary Materials

The following supporting information can be downloaded at https://www.mdpi.com/article/10.3390/ijms25126721/s1. References [122,123,124,125,126] are cited in the supplementary materials.
