# Supplementary material for: Modulation of Photosystem II Function in Celery via Foliar-Applied Salicylic Acid during Gradual Water Deficit Stress
Source: Int J Mol Sci. 2024 Jun 18;25(12):6721. doi: 10.3390/ijms25126721 (PMC11203862; doi:10.3390/ijms25126721)
Supplement: Supplementary file 1 [file ijms-25-06721-s001.zip › ijms-3043165-supplementary.pdf]

International Journal of *Molecular Sciences*

MDPI

Article

Modulation of Photosystem II Function in Celery by Foliar Applied Salicylic Acid during Gradual Water Deficit Stress

Michael Moustakas, Emmanuel Panteris, Julietta Moustaka, Tuğba Aydın, Gülriz Bayçu, and Ilektra Sperdouli

Table S1. Definitions of the chlorophyll fluorescence parameters used in the experiments

Parameter	Definition	Calculation
Φ_{PSII}	Effective quantum yield of PSII photochemistry	(F*m*′ – F*s*)/F*m*′ [61,122]
Φ_{NPQ}	Quantum yield of regulated non-photochemical energy loss in PSII	F*s*/F*m*′ – F*s*/F*m* [123]
Φ_{NO}	Quantum yield of non-regulated energy loss in PSII	F*s*/F*m* [123]
F*v*′/F*m*′	Efficiency of the open PSII reaction centers	(F*m*′ – F*o*′)/F*m*′ [122]
ETR	Electron transport rate	Φ_{PSII} × PAR × c × abs, where PAR is the photosynthetically active radiation, c is 0.5, and abs is the total light absorption of the leaf taken as 0.84 [124]
q*p*	Photochemical quenching, representing the redox state of quinone A (Q_A), or in other words the fraction of open PSII reaction centers based on the "puddle" model for the photosynthetic unit	(F*m*′ – F*s*)/(F*m*′ – F*o*′) [122]
NPQ	Non-photochemical quenching reflecting the dissipation of excitation energy as heat	(F*m* – F*m*′)/F*m*′ [125]
EXC	Excess excitation energy	(F*v*/F*m* – Φ_{PSII})/F*v*/F*m* [126]
1-qL	The fraction of closed PSII reaction centres based on the "lake" model for the photosynthetic unit	1- (q_p × F_o'/F_s) [61]
